# The HAS-BLED Score Identifies Patients with Acute Venous Thromboembolism at High Risk of Major Bleeding Complications during the First Six Months of Anticoagulant Treatment

**DOI:** 10.1371/journal.pone.0122520

**Published:** 2015-04-23

**Authors:** Judith Kooiman, Nadja van Hagen, Antonio Iglesias del Sol, Erwin V. Planken, Gregory Y. H. Lip, Felix J. M. van der Meer, Suzanne C. Cannegieter, Frederikus A. Klok, Menno V. Huisman

**Affiliations:** 1 Department of Thrombosis and Haemostasis, Leiden University Medical Center, Leiden, The Netherlands; 2 Department of Internal Medicine, Rijnland Hospital, Leiderdorp, The Netherlands; 3 Department of Internal Medicine, Diaconessenhuis, Leiden, The Netherlands; 4 University of Birmingham Centre for Cardiovascular Sciences, City Hospital, Birmingham, United Kingdom; 5 Department of Clinical Epidemiology, Leiden University Medical Center, Leiden, The Netherlands; IIBB-CSIC-IDIBAPS, SPAIN

## Abstract

**Objective:**

The HAS-BLED score enables a risk estimate of major bleeds in patients with atrial fibrillation on vitamin K-antagonists (VKA) treatment, but has not been validated for patients with venous thromboembolism (VTE). We analyzed whether the HAS-BLED score accurately identifies patients at high risk of major bleeds during VKA treatment for acute VTE.

**Methods:**

Medical records of 537 patients with acute VTE (primary diagnosis pulmonary embolism in 223, deep vein thrombosis in 314) starting VKA treatment between 2006-2007 were searched for items on the HAS-BLED score and the occurrence of major bleeds during the first 180 days of follow-up. The hazard ratio (HR) for the occurrence of major bleeds comparing non-high with high-risk patients as defined by a HAS-BLED score ≥ 3 points was calculated using Cox-regression analysis.

**Results:**

Major bleeds occurred in 11/537 patients (2.0%, 5.2/100 person years, 95% CI 2.8-9.2). Cumulative incidences of major bleeds were 1.3% (95% CI 0.1-2.5) in the non-high (HAS-BLED < 3) and 9.6% (95%CI 2.2-17.0) in the high-risk group (HAS-BLED ≥ 3), (p <0.0001 by Log-Rank test), with a HR of 8.7 (95% CI 2.7-28.4). Of the items in the HAS-BLED score, abnormal renal function (HR 10.8, 95% CI 1.9-61.7) and a history of bleeding events (HR 10.4, 95% CI 2.5-42.5) were independent predictors of major bleeds during follow-up.

**Conclusion:**

Acute VTE patients with a HAS-BLED score ≥ 3 points are at increased risk of major bleeding. These results warrant for correction of the potentially reversible risk factors for major bleeding and careful International Normalized Ratio monitoring in acute VTE patients with a high HAS-BLED score.

## Introduction

Venous thromboembolism (VTE) is the third most common cardiovascular disease affecting 1–2 per 1000 adults annually [1]. VTE requires acute treatment with low-molecular-weight-heparin followed by at least three months of therapy with oral anticoagulants, such as vitamin K-antagonists (VKA) according to the American College of Chest Physicians (ACCP) guideline [2]. Although this treatment strategy is fairly effective in preventing VTE recurrences, it causes major bleeding complications with an incidence of 2-7/100 patient years, [3–6] which are associated with increased morbidity, mortality, and health care costs [7,8].

Identifying patients at high risk of major bleeding events is therefore of importance and would help physicians targeting bleeding preventive strategies, such as adequate control of hypertension, discouraging the use of non-steroidal anti-inflammatory drugs or platelet-inhibitors, and frequent International Normalized Ratio (INR) monitoring. However, externally validated bleeding risk scores with adequate discriminative power are lacking for the VTE population [2], whereas several algorithms have been developed for patients with atrial fibrillation treated with VKA [9–12], including the HAS-BLED score (Hypertension, Abnormal renal/liver function, Stroke, Bleeding, Labile INR, Elderly, Drugs or alcohol use) [12]. The HAS-BLED score has been validated in several independent cohorts of patients with atrial fibrillation [13–15], but it is currently unknown whether the HAS-BLED score accurately predicts major bleeding events in patients with acute VTE.

The aim of our study was to analyse whether the HAS-BLED score accurately identifies patients at high risk of major bleeds during VKA treatment for acute VTE.

## Methods

We identified all patients starting VKA treatment for acute VTE (deep vein thrombosis, pulmonary embolism, or both) between January 2006 and March 2007 via records of the Leiden anticoagulation clinic. This timeframe was chosen to ensure sufficient follow-up time for included patients and treatment of acute VTE according to current clinical practice. Patients had to be treated for acute VTE by the affiliated academic or one of the two affiliated non-academic teaching hospitals of this anticoagulation clinic to be selected for inclusion for logistic reasons (Leiden University Medical Center, Leiden; Diaconessenhuis Leiden; and Rijnland hospital, Leiderdorp, all in the Netherlands). VTE diagnosis was objectified by computed tomography-pulmonary angiography or ultrasound. Patients were managed according to clinical practice with initial low-molecular-weight-heparin followed by long-term VKA therapy (either phenprocoumon or acenocoumarol). The study was approved for all three participating centers by the ethics committee of the Leiden University Medical Center, Leiden, The Netherlands (approval number P14.017/NV/ib). Patient information was anonymized prior to analysis. The need for informed consent was waived by the ethics committee.

### Chart review

Medical records from two sources (i.e. the three hospitals and the Leiden anticoagulation clinic) were searched for information on patient characteristics at baseline, INR-values, major bleeding complications, and items on the HAS-BLED score. These items were assessed at time of diagnosis of acute VTE, except for labile INR, and were scored as follows: Hypertension (i.e. systolic blood pressure > 160 mmHg) one point; Abnormal liver (history of cirrhosis, or bilirubin > 2x the upper limit of normal in association with aspartate aminotransferase/alanine aminotransferase/alkaline phosphatase levels > 3x the upper limit of normal) or renal function (on dialysis, a history of kidney transplantation, or serum creatinine values > 200 μmol/L) one point each; Stroke (history of) one point; Bleeding (history of bleeding requiring hospitalization and/or bleeding resulting in a decrease in hemoglobin level of > 2 g/L and/or bleeding requiring blood transfusion that was not a hemorrhagic stroke) one point; Labile INR during follow-up (time within therapeutic range < 60%) one point; Elderly (age > 65 years) one point; and Drugs (use of platelet inhibitors or non-steroidal anti-inflammatory drugs)/alcohol use (more than 8 units per week), one point each [12]. Laboratory measurements on renal and liver function were recorded up to six months prior to diagnosis of acute VTE, with a preference for the values closest to the day of VTE diagnosis. Missing variables on the HAS-BLED score were scored as normal (i.e. zero points), since this strategy is widely accepted in clinical application of prediction models [16–18]. INR-values were measured using HepatoQuick (Roche Diagnostics, Mannheim, Germany). Time within therapeutic range was computed using the *Rosendaal* method [19]. According to clinical practice within the Netherlands, INR target range was set for all patients between 2.5–3.5, although INR values within a therapeutic INR range of 2.0–3.5 were accepted. Time within therapeutic range was therefore computed using the therapeutic instead of the target range.

### Clinical outcome and follow-up

The primary outcome was the occurrence of major bleeding events, defined by the International Society of Thrombosis and Hemostasis (ISTH) criteria; i.e. fatal bleeding; bleeding causing a drop in hemoglobin of at least 1.24 mmol/L; or requiring transfusion of at least 2 units of whole blood or red cells; or symptomatic bleeding in a critical organ or area (i.e. intracranial, intraocular, intraspinal, retroperitoneal, intra-articular, pericardial or intramuscular with accompanying compartment syndrome) [20]. All major bleeds were independently assessed by two researchers (NvH and JK). In case of disagreements, a third researcher (MVH) was consulted. Follow-up was defined as time elapsed between VTE diagnosis and major bleeds, or death, or discontinuation of VKA therapy, with a maximum duration of 180 days.

### Statistical analyses

Incidence proportions and incidence rates of major bleeding complications with corresponding 95% confidence intervals (CI) were reported within the total population and for each outcome of the HAS-BLED score (total score ranging from zero to five points). Patients were classified as non-high or high-risk of major bleeding events based on the reported major bleeding rates for each outcome of the HAS-BLED score, with a cut-off of 7.3% as indicated by previous studies within the VTE population [18,21–24], and based on a HAS-BLED score cut-off ≥ 3 points as is used for patients with atrial fibrillation [25]. We calculated hazard ratios (HR) of major bleeds comparing high risk with non-high risk patients according to the different HAS-BLED cut-off levels. Additionally, we assessed the positive and negative predictive value, sensitivity and specificity of these HAS-BLED score cut-offs for the endpoint of major bleeds. The predictive value of the HAS-BLED score for the occurrence of major bleeds was analyzed by calculation of the C-statistic (i.e. area under the receiver operating curve) with corresponding 95% CI. We also calculated the C-statistic of the HAS-BLED score without the item of labile INR, as this information is not present at time of VKA initiation in daily practice. Independent predictors of major bleeding events were identified by the use of uni- and multivariate Cox-regression analyses, and their effect sizes were reported as hazard ratios (HR) with corresponding 95% CI. Statistical analyses were performed in SPSS 20.0 (IBM SPSS statistics, IBM Corp, Somers, NY).

## Results

In total, 700 patients started VKA therapy for acute VTE during our study period. Of those, 163 were not treated for acute VTE by one of the three affiliated hospitals and were therefore excluded. As a result, the study population comprised 537 patients, of whom 223 (41.5%) were primarily diagnosed with pulmonary embolism, and 314 (58.5%) with deep vein thrombosis. All other patient characteristics at baseline are reported in Table 1. The medical records of 388 of 537 patients lacked information on one or more items of the HAS-BLED score, most frequently on alcohol use (331/537 patients).

**Table 1:** Patient characteristics at baseline.

Characteristic	Pulmonary embolism	Deep vein thrombosis
N	223	314
Mean age (range)	60.8(81)	57.8(79)
Sex (male)	106(47.5)	150(47.8)
Prior VTE	28(12.6)	77(24.5)
Prior major bleeding^1^	7(3.1)	12(3.8)
Hypertension^2^	68(30.5)	101(32.2)
Diabetes mellitus	11(4.9)	26(8.3)
Abnormal renal function^3^	8(3.6)	3(1.0)
Abnormal liver function^4^	2(0.9)	3(1.0)
History of stroke or TIA^5^	10(4.5)	13(4.1)
Congestive heart failure	10(4.5)	6(1.9)
Idiopathic VTE	122(54.7)	151(48.1)
Active malignancy	43(19.3)	51(16.2)
Use of NSAIDs or platelet-inhibitors	30(13.5)	30(9.6)
Other vascular disease	25(11.2)	20(6.4)
Prior myocardial infarction	16(7.2)	16(5.1)
Peripheral artery disease	7(3.1)	4(1.3)
Both	2(0.9)	0(0.0)
Alcohol use > 8 units per week^6^	19(8.5)	29(9.2)

Data are presented as n, % unless stated otherwise

Abbreviations: VTE = venous thromboembolism, NSAIDS = non-steroidal anti-inflammatory drugs, TIA = transient ischemic attack, INR = international normalized ratio

^1^Unknown in 39 patients,

^2^Blood pressure measurements missing in 169 patients,

^3^information on renal function lacking in 114 patients,

^4^Information on liver function lacking in 127 patients,

^5^28 patients lacking information on previous stroke or TIA,

^6^Unknown in 331 patients

Follow-up on anticoagulant treatment was complete for all patients. Median duration of follow-up was 179 days (2.5–97.5 percentiles 13–180 days). In total, 43 patients (8.0%) died during follow-up, of whom 21 (48.8%) of malignancy. Median time within therapeutic range during the entire duration of follow-up was 78.2% (95% CI 23.8–99.9%), and 21.6% (116/537) of patients had a labile INR according to the definition of the HAS-BLED score (i.e. time within therapeutic range—< 60%).

### Major bleeding

During 180 days follow-up, 11/537 patients (2.0%; 5.2/100 person years, 95% CI 2.8–9.2) developed a major bleeding event. Median time to the occurrence of bleeding in those 11 patients was 61 days (min 6, max 148 days). Three (27.3%) of eleven bleeds were gastrointestinal, three (27.3%) intramuscular, one (9.1%) retroperitoneal, and four (36.4%) at other locations. Bleeding was fatal in none of the eleven patients experiencing a major bleeding complication. Mean INR during follow-up was 2.9 (SD 1.1) for patients developing a major bleeding event and 2.8 (SD 0.9) for those who did not (p 0.12).

### Test characteristics of the HAS-BLED score

**Fig 1:**
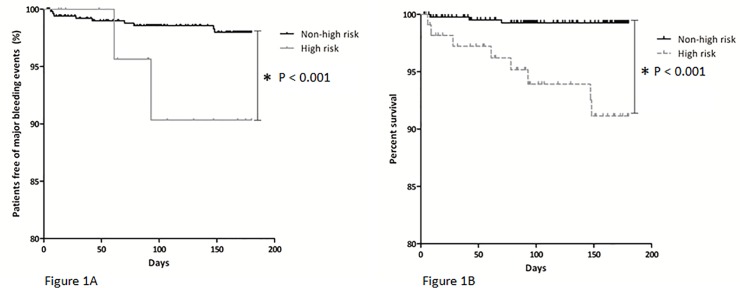
Percent survival of major bleeding complications by Kaplan-Meier life table method, stratified to A) non-high (HAS-BLED score < 4) or high-risk (HAS-BLED score ≥ 4) of major bleeds; * p = 0.0007 by Log-Rank test, HR of 10.8 (95% CI 2.3–50.0) or B) non-high (HAS-BLED score < 3) or high-risk (HAS-BLED score ≥ 3) of major bleeds; * p <0.0001 by Log-Rank test, HR of 8.7 (95%CI2.7–28.4).

When high-risk of major bleeds was defined by a HAS-BLED score of 3 points or higher as is used for patients with atrial fibrillation, 13.6% (73/537) of patients were identified as high-risk. Cumulative incidences of major bleeds were 1.3% (95%CI 0.1–2.5) in the non-high and 9.6% (95%CI 2.2–17.0) in the high-risk group (p <0.0001 by Log-Rank test), which resulted in a HR for major bleeds of 8.7 (95%CI2.7–28.4) in high-risk patients (Fig 1).

According to the predefined major bleeding risk cut-off of 7.3% for the definition of high-risk as indicated by previous studies within the VTE population[18,21–24], patients with a HAS-BLED score of 4 (instead of 3) points or higher were classified as high-risk of major bleeding events (Table 2). The HAS-BLED score categorized 15/537 (2.8%) patients as high-risk of bleeding using this cut-off level. Two out of eleven patients (18.2%) who developed a major bleeding event were identified as high-risk by this cut-off point. The cumulative incidences of major bleeds were 2.0% (95%CI 0.6–3.4) in the non-high and 22.1% (95%CI 0.0–49.7) in the high-risk group, (p<0.001 by Log-Rank test, Fig 1), with a HR of 10.8 (95% CI 2.3–50.0) for major bleeding in high-risk patients.

**Table 2:** Risk of major bleeding events during 180 days follow-up stratified to the HAS-BLED score.

HAS-BLED				
Score	No.	No. bleeds	Incidence proportion	95% CI
0	163	0	0.0	(0.0–2.8)
1	172	3	1.7	(0.4–5.2)
2	129	2	1.6	(0.1–5.8)
3	59	4	6.8	(2.2–16.6)
≥ 4	14	2	14.3	(2.8–41.2)

Incidence proportions presented as percentages

For both cut-offs on the HAS-BLED score, we reported the positive and negative predictive value, sensitivity and specificity for the endpoint of major bleeds in Table 3.

**Table 3:** Test characteristics of the HAS-BLED score at two different cut-off levels.

HAS-BLED cut-off	Positive predictive value (%, 95%CI)	Negative predictive value (%, 95%CI)	Sensitivity (%, 95% CI)	Specificity (%, 95% CI)	Hazard ratio* (95% CI)			
≥ 3	8.2 (3.1–17.0)	98.9 (97.5–99.7)	54.6 (23.5–83.1)	87.3 (84.1–90.0)	8.7	(2.6	-	28.4)
≥ 4	14.3 (2.2–42.8)	98.3 (96.8–99.2)	18.2 (2.8–51.8)	97.7 (96.1–98.8)	10.8	(2.3	-	50.0)

* Crude hazard ratio for major bleeding events during 180 days of follow-up

The C-statistic of the HAS-BLED score for the prediction of major bleeds was 0.78 (95% CI 0.65–0.91). Excluding the items ‘labile INR’ or ‘alcohol use’ (as information on this item was missing in 331/537 patients) of the HAS-BLED score resulted in C-statistics of 0.81 (95% CI 0.70–0.92) and 0.81 (95% CI 0.71–0.91), respectively.

### Risk factors for major bleeds

Of the items in the HAS-BLED score, abnormal renal function and a history of bleeding events were independent predictors of major bleeds during follow-up with HRs of 10.8 (95% CI 1.9–61.7) and 10.4 (95% CI 2.5–42.5), respectively (Table 4).

**Table 4:** Uni- and multivariate Cox-regression analyses.

	Univariate analysis					Multivariate analysis				
	Regression coefficient	HR	95% CI			Regression coefficient	HR	95% CI		
***HAS-BLED items***			** **					** **		
Hypertension	1.8	6.0	(1.6	-	22.8)	1.0	2.7	(0.6	-	11.6)
Abnormal kidney function	2.5	12.3	(2.6	-	56.8)	2.4	10.8	(1.9	-	61.7)
Abnormal liver function	NA			-		NA			-	
Stroke in history	NA			-		NA			-	
Bleeding in history	2.5	12.4	(3.3	-	47.0)	2.3	10.4	(2.5	-	42.5)
Labile INR	0.5	1.6	(0.4	-	6.0)	0.4	1.4	(0.4	-	5.7)
Elderly	1.4	4.1	(1.1	-	15.4)	1.3	3.7	(0.8	-	16.2)
Drugs	0.3	1.3	(0.2	-	10.4)	- 0.8	0.4	(0.0	-	4.4)
Alcohol	-0.0	1.0	(0.1	-	7.7)	NA			-	

Abbreviations: HR = hazard ratio, CI = confidence interval, NA = not applicable

Definitions: Hypertension = systolic blood pressure > 160 mmHg; Abnormal liver function = history of cirrhosis, or bilirubin > 2x the upper limit of normal in association with aspartate aminotransferase/alanine aminotransferase/alkaline phosphatase levels > 3x the upper limit of normal; Abnormal renal function = on dialysis, a history of kidney transplantation, or serum creatinine values > 200 μmol/L; Labile INR = time within therapeutic range < 60%; Elderly = age > 65 years; Drugs = use of platelet inhibitors or non-steroidal anti-inflammatory drugs/alcohol use (more than 8 units per week)

## Discussion

We aimed to evaluate whether the HAS-BLED score predicts major bleeding complications in patients with acute VTE during VKA therapy. Our study demonstrates that patients with a HAS-BLED score ≥ 3 points are at 8-fold increased risk of major bleeding complications during the first 180 days of VKA treatment. However, despite a good specificity and negative predictive value, the sensitivity of the HAS-BLED score at this cut-off was only 54.6%. As a result, the HAS-BLED score might not be useful in identifying patients at truly high risk of major bleeding during VKA therapy for acute VTE.

Risk stratification has emerged as an important tool for both patient-level decision making and risk assessment and adjustment to improve quality of care. Over the last two decades, several attempts have been made to develop a proper algorithm to estimate the risk of major bleeding events during anticoagulant treatment for acute VTE [21,22,24,26]. Although the RIETE, Kuijer, Kearon, and OBRI scores all reported promising results in their derivation and internal validation studies [21,22,24,26], their predictive value was reported poor by external validation cohorts, with c-statistics ranging between 0.28 and 0.60 [18,27].

The HAS-BLED score has shown to be of predictive value for major bleeds in several external validation cohorts of patient with atrial fibrillation treated with VKAs [15,28–30], but also in cohorts of patients with other indications for the use of anticoagulants [31–33]. Three previous studies analyzed the predictive value of the HAS-BLED score in VTE patients. The first study was performed in elderly patients (i.e. age > 80 years) and found C-statistic of 0.55, probably among others due to that the HAS-BLED variable ‘elderly’ (i.e. age > 65 years) has no discriminative power in this population [34]. Two other studies performed in patients with divergent indications for VKA use, such as VTE and atrial fibrillation, reported a C-statistics of 0.57 and 0.67 for the HAS-BLED score for the entire populations, without reporting these figures for the VTE population separately [27,35]. As neither of these studies reported test characteristics of the HAS-BLED score in the general VTE population, their results are hard to translate into clinical practice.

Based on previous studies on major bleeding risks in VTE patients and the incidences found in our study, patients with a HAS-BLED score of four or higher can be regarded as high risk [18,21–24]. However, for the atrial fibrillation population a HAS-BLED score of three or higher is regarded as high-risk of major bleeds [25]. Both cut-offs demonstrated to be predictive of major bleeding events during follow-up with a HR of 8.7 for the cut-off of three points or higher and HR 10.8 for the cut-off of at least four points. Nevertheless, 4 of 11 patients with a major bleed during follow-up had an HAS-BLED score of 3 and would have been missed by a HAS-BLED score cut-off of four points or higher, which resulted in a low sensitivity (18%). We therefore regard the use of the cut-off of 3 points or higher more useful for the identification of high risk patients, although this might result in a lower specificity (97.7% vs 87.3%).

Implementation of the HAS-BLED score in clinical management of patients with acute VTE should be done with caution. Although a HAS-BLED score of 3 points or higher was shown to be a good predictor and of high specificity for major bleeds in our study, the sensitivity at this cut-off was only 54.6% with a positive predictive value of 8.2%. This means that 92% of patients that are identified as high risk of bleeding during VKA therapy will not develop this event. So how could the HAS-BLED score potentially be used in daily practice of patients with acute VTE? We emphasize that, regardless of the predictive value of any bleeding score, withholding anticoagulants is unacceptable in these patients, including those with a high bleeding risk. However, we do advocate that physicians take appropriate bleeding preventive measures in patients at high risk of bleeding according to the HAS-BLED score, to correct the potentially reversible risk factors such as adequate control of blood pressure, frequent INR monitoring, and withholding non-steroidal anti-inflammatory drugs or platelet-inhibitors. Moreover, patient education about anticoagulant treatment, coaching and self-monitoring of INR values may also help to prevent major bleeding complications in these patients, although the effect of patient education on clinical outcomes remains unclear [36] [37]. Whether the HAS-BLED score can also be used to predict bleeding complications during extended VKA treatment for VTE, or during therapy with any of the novel oral anticoagulants can’t be studied in our cohort, and should be focus of future research.

Our study adds clinically relevant information to the research field as we analysed the discriminative value of a simple algorithm for the endpoint of major bleeds, of which the items are readily available in daily practice. However, some aspects of our study warrant comment. First, due to its retrospective design, 388 of 537 patients had missing information on one or more items of the HAS-BLED score, most frequently on alcohol use (lacking in 331 patients). These elements were analyzed as normal (i.e. zero points), as indicated by previous studies [16–18]. This could have resulted in an underestimation of a patient’s bleeding risk as assessed by the HAS-BLED score. Importantly, sensitivity analyses excluding either patients with missing items or including all patients but excluding the item of alcohol use on the HAS-BLED score demonstrated similar results on the discriminative value of this bleeding score (data not shown). Second, we cannot exclude that some major bleeding events were missed, as we based our results on information available in medical records at the participating hospitals and anticoagulation clinic. However, major bleeds are serious medical events leading to evaluation in a hospital setting and thus unlikely to be missed in medical records. Moreover, our reported major bleeding incidence rate of 5/100 person years compares well to the existing literature [3–6], which makes it unlikely that events were missed. Third, the number of major bleeding events in our cohort was limited (i.e. eleven). It would therefore be valuable if the results of our study would be confirmed by larger cohorts of acute VTE patients. Fourth, we excluded 163 of 700 patients as they had not been treated for their acute VTE event by one of the three designated hospitals. However, it is unlikely that this would have had an impact on the discriminative value of the HAS-BLED score, as this exclusion criterion was solely based on geographics and not on relevant patient characteristics. Fifth, we did not perform a formal power calculation. However, our sample size is in line with that of previous validation studies on bleeding risk scores in the acute VTE population [18,27,35,38].

In conclusion, patients with acute VTE and a HAS-BLED score of three points or higher are at high risk of major bleeding events during anticoagulant treatment. These results warrant for correction of the potentially reversible risk factors for major bleeding and careful INR monitoring in acute VTE patients with a high HAS-BLED score.

## Supporting Information

S1 Database HAS-BLED database. (SAV)

## References

[1] Oger E (2000) Incidence of venous thromboembolism: a community-based study in Western France. EPI-GETBP Study Group. Groupe d'Etude de la Thrombose de Bretagne Occidentale. Thromb Haemost 83: 657–660. 00050657 [pii]. 10823257

[2] Kearon C, Akl EA, Comerota AJ, Prandoni P, Bounameaux H, Goldhaber SZ, et al (2012) Antithrombotic therapy for VTE disease: Antithrombotic Therapy and Prevention of Thrombosis, 9th ed: American College of Chest Physicians Evidence-Based Clinical Practice Guidelines. Chest 141: e419S–e494S. 141/2_suppl/e419S [pii]; 10.1378/chest.11-2301 22315268 PMC3278049

[3] Kearon C, Gent M, Hirsh J, Weitz J, Kovacs MJ, Anderson DR, et al (1999) A comparison of three months of anticoagulation with extended anticoagulation for a first episode of idiopathic venous thromboembolism. N Engl J Med 340: 901–907. 10.1056/NEJM199903253401201 10089183

[4] Linkins LA, Choi PT, Douketis JD (2003) Clinical impact of bleeding in patients taking oral anticoagulant therapy for venous thromboembolism: a meta-analysis. Ann Intern Med 139: 893–900. 139/11/893 [pii]. 14644891 10.7326/0003-4819-139-11-200312020-00007

[5] Schulman S, Beyth RJ, Kearon C, Levine MN (2008) Hemorrhagic complications of anticoagulant and thrombolytic treatment: American College of Chest Physicians Evidence-Based Clinical Practice Guidelines (8th Edition). Chest 133: 257S–298S. 133/6_suppl/257S [pii]; 10.1378/chest.08-0674 18574268

[6] Schulman S, Granqvist S, Holmstrom M, Carlsson A, Lindmarker P, Nicol P, et al (1997) The duration of oral anticoagulant therapy after a second episode of venous thromboembolism. The Duration of Anticoagulation Trial Study Group. N Engl J Med 336: 393–398. 10.1056/NEJM199702063360601 9010144

[7] Nieto JA, Camara T, Gonzalez-Higueras E, Ruiz-Gimenez N, Guijarro R, Marchena PJ, et al(2008) Clinical outcome of patients with major bleeding after venous thromboembolism. Findings from the RIETE Registry. Thromb Haemost 100: 789–796. 08110789 [pii]. 18989522

[8] White RH, Beyth RJ, Zhou H, Romano PS (1999) Major bleeding after hospitalization for deep-venous thrombosis. Am J Med 107: 414–424. S0002-9343(99)00267-3 [pii]. 10569295 10.1016/s0002-9343(99)00267-3

[9] Fang MC, Go AS, Chang Y, Borowsky LH, Pomernacki NK, Udaltsova N, et al (2011) A new risk scheme to predict warfarin-associated hemorrhage: The ATRIA (Anticoagulation and Risk Factors in Atrial Fibrillation) Study. J Am Coll Cardiol 58: 395–401. S0735-1097(11)01571-3 [pii]; 10.1016/j.jacc.2011.03.031 21757117 PMC3175766

[10] Gage BF, Yan Y, Milligan PE, Waterman AD, Culverhouse R, Rich MW, et al (2006) Clinical classification schemes for predicting hemorrhage: results from the National Registry of Atrial Fibrillation (NRAF). Am Heart J 151: 713–719. S0002-8703(05)00436-9 [pii]; 10.1016/j.ahj.2005.04.017 16504638

[11] Shireman TI, Mahnken JD, Howard PA, Kresowik TF, Hou Q, Ellerbeck EF (2006) Development of a contemporary bleeding risk model for elderly warfarin recipients. Chest 130: 1390–1396. 130/5/1390 [pii]; 10.1378/chest.130.5.1390 17099015

[12] Pisters R, Lane DA, Nieuwlaat R, de Vos CB, Crijns HJ, Lip GY (2010) A novel user-friendly score (HAS-BLED) to assess 1-year risk of major bleeding in patients with atrial fibrillation: the Euro Heart Survey. Chest 138: 1093–1100. chest.10-0134 [pii]; 10.1378/chest.10-0134 20299623

[13] Apostolakis S, Lane DA, Guo Y, Buller H, Lip GY (2013) Performance of the HEMORR 2 HAGES, ATRIA, and HAS-BLED bleeding risk-prediction scores in nonwarfarin anticoagulated atrial fibrillation patients. J Am Coll Cardiol 61: 386–387. S0735-1097(12)05300-4 [pii]; 10.1016/j.jacc.2012.10.010 23246385

[14] Lip GY, Lin HJ, Hsu HC, Su TC, Chen MF, Lee YT, et al (2013) Comparative assessment of the HAS-BLED score with other published bleeding risk scoring schemes, for intracranial haemorrhage risk in a non-atrial fibrillation population: The Chin-Shan Community Cohort Study. Int J Cardiol. S0167-5273(12)01705-6 [pii]; 10.1016/j.ijcard.2012.12.076 23336959

[15] Apostolakis S, Lane DA, Guo Y, Buller H, Lip GY (2012) Performance of the HEMORR(2)HAGES, ATRIA, and HAS-BLED bleeding risk-prediction scores in patients with atrial fibrillation undergoing anticoagulation: the AMADEUS (evaluating the use of SR34006 compared to warfarin or acenocoumarol in patients with atrial fibrillation) study. J Am Coll Cardiol 60: 861–867. S0735-1097(12)02368-6 [pii]; 10.1016/j.jacc.2012.06.019 22858389

[16] Aujesky D, Obrosky DS, Stone RA, Auble TE, Perrier A, Cornuz J, et al (2005) Derivation and validation of a prognostic model for pulmonary embolism. Am J Respir Crit Care Med 172: 1041–1046. 200506-862OC [pii]; 10.1164/rccm.200506-862OC 16020800 PMC2718410

[17] Fine MJ, Auble TE, Yealy DM, Hanusa BH, Weissfeld LA, Singer DE, et al (1997) A prediction rule to identify low-risk patients with community-acquired pneumonia. N Engl J Med 336: 243–250. 10.1056/NEJM199701233360402 8995086

[18] Scherz N, Mean M, Limacher A, Righini M, Jaeger K, Beer HJ, et al (2013) Prospective, multicenter validation of prediction scores for major bleeding in elderly patients with venous thromboembolism. J Thromb Haemost 11: 435–443. 10.1111/jth.12111 23279158

[19] Rosendaal FR, Cannegieter SC, van der Meer FJ, Briet E (1993) A method to determine the optimal intensity of oral anticoagulant therapy. Thromb Haemost 69: 236–239. 8470047

[20] Schulman S, Kearon C (2005) Definition of major bleeding in clinical investigations of antihemostatic medicinal products in non-surgical patients. J Thromb Haemost 3: 692–694. JTH1204 [pii]; 10.1111/j.1538-7836.2005.01204.x 15842354

[21] Kuijer PM, Hutten BA, Prins MH, Buller HR (1999) Prediction of the risk of bleeding during anticoagulant treatment for venous thromboembolism. Arch Intern Med 159: 457–460. 10074953 10.1001/archinte.159.5.457

[22] Kearon C, Ginsberg JS, Kovacs MJ, Anderson DR, Wells P, Julian JA, et al (2003) Comparison of low-intensity warfarin therapy with conventional-intensity warfarin therapy for long-term prevention of recurrent venous thromboembolism. N Engl J Med 349: 631–639. 10.1056/NEJMoa035422 ;349/7/631 [pii]. 12917299

[23] Ruiz-Gimenez N, Suarez C, Gonzalez R, Nieto JA, Todoli JA, Samperiz AL, et al (2008) Predictive variables for major bleeding events in patients presenting with documented acute venous thromboembolism. Findings from the RIETE Registry. Thromb Haemost 100: 26–31. 08070026 [pii]; 10.1160/TH08-03-0193 18612534

[24] Beyth RJ, Quinn LM, Landefeld CS (1998) Prospective evaluation of an index for predicting the risk of major bleeding in outpatients treated with warfarin. Am J Med 105: 91–99. S0002934398001983 [pii]. 9727814 10.1016/s0002-9343(98)00198-3

[25] Camm AJ, Lip GY, De CR, Savelieva I, Atar D, Hohnloser SH, et al (2012) 2012 focused update of the ESC Guidelines for the management of atrial fibrillation: an update of the 2010 ESC Guidelines for the management of atrial fibrillation—developed with the special contribution of the European Heart Rhythm Association. Europace 14: 1385–1413. eus305 [pii]; 10.1093/europace/eus305 22923145

[26] Ruiz-Gimenez N, Suarez C, Gonzalez R, Nieto JA, Todoli JA, Samperiz AL, et al (2008) Predictive variables for major bleeding events in patients presenting with documented acute venous thromboembolism. Findings from the RIETE Registry. Thrombosis and Haemostasis 100: 26–31. 10.1160/TH08-03-0193 18612534

[27] Donze J, Rodondi N, Waeber G, Monney P, Cornuz J, Aujesky D (2012) Scores to predict major bleeding risk during oral anticoagulation therapy: a prospective validation study. Am J Med 125: 1095–1102. S0002-9343(12)00287-2 [pii]; 10.1016/j.amjmed.2012.04.005 22939362

[28] Seet RC, Rabinstein AA, Christianson TJ, Petty GW, Brown RD Jr. (2013) Bleeding complications associated with warfarin treatment in ischemic stroke patients with atrial fibrillation: a population-based cohort study. J Stroke Cerebrovasc Dis 22: 561–569. S1052-3057(13)00025-6 [pii]; 10.1016/j.jstrokecerebrovasdis.2013.01.019 23499334 PMC4080718

[29] Gallego P, Roldan V, Torregrosa JM, Galvez J, Valdes M, Vicente V, et al (2012) Relation of the HAS-BLED bleeding risk score to major bleeding, cardiovascular events, and mortality in anticoagulated patients with atrial fibrillation. Circ Arrhythm Electrophysiol 5: 312–318. CIRCEP.111.967000 [pii]; 10.1161/CIRCEP.111.967000 22319005

[30] Lip GY, Frison L, Halperin JL, Lane DA (2011) Comparative validation of a novel risk score for predicting bleeding risk in anticoagulated patients with atrial fibrillation: the HAS-BLED (Hypertension, Abnormal Renal/Liver Function, Stroke, Bleeding History or Predisposition, Labile INR, Elderly, Drugs/Alcohol Concomitantly) score. J Am Coll Cardiol 57: 173–180. S0735-1097(10)04337-8 [pii]; 10.1016/j.jacc.2010.09.024 21111555

[31] Lip GY, Lin HJ, Hsu HC, Su TC, Chen MF, Lee YT, et al (2013) Comparative assessment of the HAS-BLED score with other published bleeding risk scoring schemes, for intracranial haemorrhage risk in a non-atrial fibrillation population: The Chin-Shan Community Cohort Study. Int J Cardiol. S0167-5273(12)01705-6 [pii]; 10.1016/j.ijcard.2012.12.076 23336959

[32] Smith JG, Wieloch M, Koul S, Braun OO, Lumsden J, Rydell E, et al (2012) Triple antithrombotic therapy following an acute coronary syndrome: prevalence, outcomes and prognostic utility of the HAS-BLED score. EuroIntervention 8: 672–678. EIJV8I6A105 [pii]; 10.4244/EIJV8I6A105 23086784

[33] Omran H, Bauersachs R, Rubenacker S, Goss F, Hammerstingl C (2012) The HAS-BLED score predicts bleedings during bridging of chronic oral anticoagulation. Results from the national multicentre BNK Online bRiDging REgistRy (BORDER). Thromb Haemost 108: 65–73. 11-12-0827 [pii]; 10.1160/TH11-12-0827 22534746

[34] Poli D, Antonucci E, Testa S, Cosmi B, Palareti G, Ageno W (2013) The predictive ability of bleeding risk stratification models in very old patients on vitamin K antagonist treatment for venous thromboembolism: results of the prospective collaborative EPICA study. J Thromb Haemost 11: 1053–1058. 10.1111/jth.12239 23578305

[35] Burgess S, Crown N, Louzada ML, Dresser G, Kim RB, Lazo-Langner A (2013) Clinical performance of bleeding risk scores for predicting major and clinically relevant non-major bleeding events in patients receiving warfarin. J Thromb Haemost 11: 1647–1654. 10.1111/jth.12352 23848301

[36] Wong PY, Schulman S, Woodworth S, Holbrook A (2013) Supplemental patient education for patients taking oral anticoagulants: systematic review and meta-analysis. J Thromb Haemost 11: 491–502. 10.1111/jth.12107 23279062

[37] Beyth RJ, Quinn L, Landefeld CS (2000) A multicomponent intervention to prevent major bleeding complications in older patients receiving warfarin. A randomized, controlled trial. Ann Intern Med 133: 687–695. 200011070–00010 [pii]. 11074901 10.7326/0003-4819-133-9-200011070-00010

[38] Wells PS, Forgie MA, Simms M, Greene A, Touchie D, Lewis G, et al (2003) The outpatient bleeding risk index: validation of a tool for predicting bleeding rates in patients treated for deep venous thrombosis and pulmonary embolism. Arch Intern Med 163: 917–920. 10.1001/archinte.163.8.917 ;163/8/917 [pii]. 12719200

